# The five self-harm behavior groupings measure: empirical and thematic data from a novel comprehensive self-harm assessment

**DOI:** 10.3389/fpsyt.2023.1147206

**Published:** 2023-05-05

**Authors:** Sophie I. Liljedahl, Daiva Daukantaitė, Nikolaus Kleindienst, Margit Wångby-Lundh, Sofie Westling

**Affiliations:** ^1^Department of Psychiatry for Affective Disorders, National Specialized Medical Care Unit for Severe Self-Harm Behaviour, Sahlgrenska University Hospital, Gothenburg, Sweden; ^2^Department of Psychiatry and Neurochemistry, Institute of Neuroscience and Physiology, Sahlgrenska Academy, University of Gothenburg, Gothenburg, Sweden; ^3^Finjagården Treatment Center, Finja, Sweden; ^4^Department of Psychology, Lund University, Lund, Sweden; ^5^Central Institute of Mental Health, Department of Psychosomatic Medicine and Psychotherapy, Medical Faculty Mannheim, Heidelberg University, Mannheim, Germany; ^6^Office for Psychiatry and Habilitation, Psychiatric Clinic Lund, Lund, Sweden; ^7^Department of Clinical Sciences Malmö, Psychiatry, Lund University, Lund, Sweden

**Keywords:** self-harm, 5S-HM, unified theoretical model of self-harm, sexual self-harm, indirect self-harm, harmful self-neglect

## Abstract

**Introduction:**

The Five Self-Harm Behavior Groupings Measure (5S-HM) is a novel assessment that evaluates behaviours which may go undetected by existing measures. Self-harm is formulated across directness and lethality spectra, including under-studied behaviors such as indirect self-harm, harmful self-neglect and sexual self-harm. Aims of the study were to: (1) empirically evaluate the 5S-HM; (2) to determine whether the 5S-HM generates relevant new information with respect to the forms and functions given by participants for self-harm within a clinical sample; (3) to test the utility and novel contributions of the Unified Model of Self-Harm and the 5S-HM by extension.

**Methods:**

Data were collected from *N* = 199 individuals (M_*age*_ = 29.98, SD = 8.41, 86.4% female), receiving specialized evidence-based treatments for self-harm, borderline personality disorder or eating disorders. Construct validity was determined via Spearman correlations, and internal consistency was established from Cronbach’s alpha. Inductive thematic analysis was used to analyze and interpret qualitative data on reasons, forms and functions participants reported in relation to self-harm following Braun and Clarke’s analytic guidelines. Thematic mapping was used to summarize qualitative data.

**Results:**

Test-retest reliability on a subsample of *n* = 24, tested 14 days after Time 1 was supported by a good intraclass correlation (0.68). Internal consistency (Cronbach’s alpha = 0.75) was acceptable to good, as was construct validity comparing the 5S-HM total score to two validated self-harm measures (rho = 0.40, *p* < 0.01; rho = 0.26, *p* < 0.01). A thematic map depicting antecedents and consequences of self-harm over time suggests that self-harm is initiated by negative emotional states and self-intolerance. Novel findings in relation to sexual self-harm indicated that reasons for these behaviors were either to improve or worsen one’s situation through being hurt by someone else.

**Discussion:**

The empirical analyses of the 5S-HM demonstrate that it is a robust measure for use in clinical and research settings. Thematic analyses proposed explanations for why self-harm behaviors are initiated and how they are reinforced over time. Sexual self-harm in particular requires further careful study.

## Introduction

Self-harm is common in both adolescents (1) and adults diagnosed with psychiatric disorders (2, 3). Measurement instruments exist that query non-suicidal self-injury (NSSI) (4) as well as self-harm without specifying suicidality (5). Other measures assess suicide attempts and intentional self-injury (6) the functions NSSI and self-harm serve (7, 8), and motivations for suicide attempts (9). Many specific self-harm measures exist, but a comprehensive assessment of both direct and indirect self-harm useful to clinicians and researchers alike is missing from the existing literature.

To understand self-harm in an effort at eliminating it effectively, predictors and correlates of past behaviors have been evaluated. A recent meta-analysis of 365 studies conducted over 50 years concluded that previous suicidal thoughts and behaviors (STBs) are not robust risk factors for future STBs (10). Similar findings were reported in a meta-analysis of self-injurious thoughts and behaviors (SITBs), whereby previous SITBs were described as generating diagnostic accuracy estimates that were only slightly better than chance (11). The authors encouraged forthcoming research to focus efforts at addressing gaps in many areas of the literature, including assessment as well as mechanisms driving SITBs.

With respect to clinical populations for whom self-harm may be particularly relevant, it is worth noting that recurrent self-harming behavior is a diagnostic criteria of borderline personality disorder (BPD) (12). Within clinical samples, individuals diagnosed with BPD are often studied in relation to self-harm. A leading evidence-based treatment for BPD is dialectical behavior therapy (DBT) (13, 14). Although individuals diagnosed with BPD receiving DBT complete weekly diary cards that query self-harm (15, 16), some forms of self-harming behavior may go undetected or change form over time. As such they may escape the notice of weekly diary cards that are anchored for weeks or months between revisions of therapy contracts (15). Further, sexual self-harm (17) and indirect self-harm (18) are more recently recognized forms of self-harm that may be difficult to recognize compared to readily identified behaviors such as cutting. Even though awareness exists that self-harm repertoires may change over time (16) it is not feasible to regularly dedicate session time to query possible new forms of self-harming behavior. To the best of our knowledge at the time of writing this paper, no existing assessment measure systematically evaluates self-harm behavior on a spectrum from low to high lethality (e.g., from NSSI to suicide attempt) while also evaluating indirect self-harm behaviors such as putting oneself in harms’ way as well as sexual self-harm.

The five self-harm behavior groupings measure (5S-HM) is a unified measure of self-harm that may help clinicians support the people they treat in therapy to better recognize and understand how and why self-harm develops and is maintained, both episodically and over time. This in turn may help to refine therapy as indicated by forms of self-harm and reasons for self-harming particular to the person, increasing the possibility for individualized, specialized treatment. Data from the 5S-HM may be used in clinical research to capture all possible forms of self-harming behavior within a single measure. This is desirable because it reduces participant burden in filling out multiple overlapping measures. Further, there is room to query meaning and purpose of self-harm in the section on “reasons” following each type of behavior grouping, facilitating in-depth analysis of events and behaviors that are not fully understood in self-harm research, such as indirect and sexual self-harm. These features of the 5S-HM are particularly relevant in populations for whom self-harm is frequent and severe, as measures developed for non-clinical populations may lack accuracy and reliability when administered in clinical populations (19).

The unified theoretical framework of self-harming behavior (herein, the “Unified Model”) was developed by Liljedahl and Westling (20) to have a framework for exhaustively querying all forms of self-harming behavior and to provide an assessment measure for researchers and clinicians to do so. We proposed that an accurate assessment of mental health functioning amongst self-harming individuals can only be arrived at by effectively capturing self-harm in all its various forms, while also considering changes in the forms and functions of self-harming behavior over time.

Within the Unified Model we proposed that there are common features between direct and indirect forms of self-harm, as there are between NSSI and suicide attempts (which are endpoints under the spectrum of lethality within the Unified Model) (20). Common features relate to functions of self-harm, which may influence the expression of the behavior. Functions can change over time based on emotional and cognitive state, learning and intention, clinical improvement or worsening as well as circumstances such as possible interruption at the time of self-harm (12, 20). In other words, changes in directness of the form of self-harming behavior may be distinct and meaningful clinically but are not formulated as separate or outside of a broader self-harm umbrella.

Specific common features are most apparent from reviewing the reasons people give for engaging in various forms of self-harm. Amongst individuals who use self-harm to regulate affect during times of crisis it is expected that the form of self-harm will change based on circumstances, along with associated changes related to function, directness of self-harm, as well as lethality. Suicidal intent is understood within the Unified Model as situational, fluctuating, or persistent, although not perfectly aligned to actual self-harming behavior. This is due to the high probability of cognitive and emotional dysregulation at times of crisis that makes planning behavior and articulating recall of the intention behind one’s plans particularly difficult.

The Unified Model is named for the intention to consolidate various forms of self-harm evaluations into a single measure for ease of implementation in clinical settings and as a time-effective resource for tracking changes in forms and functions of self-harm over time for clinicians and researchers alike. Based on the Unified Model (20), we developed the 5S-HM from integrating the findings from the suicide, self-harm and BPD literatures, unifying the assessment of self-harm and suicidal behaviors that previous measures have primarily assessed separately. We present the reasons people give for engaging in different forms of self-harming behavior as an initial step toward understanding how and why some behaviors emerge, and what consequences are associated with these behaviors over time, alongside empirical features of the 5S-HM. The five domains of self-harming behavior measured by the five self-harm behavior groupings measure (5S-HM: see Supplementary File 1) range from low to high lethality, with a distinction for directness to indicate whether the behavior results in immediate tissue damage. Specifically, these domains are: 1. Direct: self-injury (e.g., NSSI); 2. Indirect: harmful self-neglect (e.g., not taking prescribed medication); 3. Indirect: sexual self-harm or self-exploitation (e.g., behaviors engaged in for the purpose of harming oneself, in the absence of sexual interest, desire or curiosity); 4a. Indirect: putting oneself in harms’ way (e.g., walking alone in neighborhoods known to be unsafe due to high criminality as a method of exposing oneself to risk), 4b. Direct: putting oneself in harms’ way: (e.g., trying to light oneself on fire), 5. Direct: suicide attempt, defined as self-initiated behaviors undertaken to kill oneself with higher lethality than the direct self-injury of NSSI.

Responses to these items were weighted so that frequent and severe behaviors have a higher score. The weighting scheme was determined for each individual item of the 5S-HM collaboratively between the first and last authors, who both have significant clinical experience working with this population.

We turn our focus to the clinical phenomenon of indirect self-harm, as it has been less intensively studied in self-harm research compared to NSSI and suicidal behaviors. Given the relative research novelty of indirect self-harm, putting oneself in harms’ way and sexual self-harm, the following sections are dedicated to a summary of the research generated upon each of these phenomena. A distinction between direct and indirect forms of NSSI has been suggested (18) but has been systematically investigated less often than studies evaluating direct forms self-harm such as NSSI and suicide attempts.

A study comprised of predominantly male (78.2%) veterans from the United States evaluated direct and indirect forms of self-harm in a measure created for this purpose and population, the direct and indirect self-harm inventory (DISH) (21). Evaluated alongside a well-validated measure of self-harm, the self-injurious thoughts and behaviors interview (SITBI) (22), the DISH was better able to detect indirect self-harm, such as provoking physical fights and punching walls, as well as high-risk behaviors such as unsafe sex and reckless driving. The authors formulated self-harm as existing on a continuum ranging from low to high-risk behaviors and indirect self-harm to direct self-harm. They note that although indirect self-harm is often less severe than other forms of direct self-harm behaviors, it is more likely to accumulate, with harm to the individual going clinically undetected (21). Accordingly, we assess indirect self-harm in the 5S-HM.

Indirect behaviors that are not well investigated in the literature is harmful self-neglect or putting oneself in harms’ way, directly or indirectly. These behaviors can be indirect, such as walking into traffic without checking for safety, provoking physical fights for the purpose of exposing oneself to harm, walking alone in neighborhoods known to be dangerous or affiliating with gang members for the purpose of exposing oneself to harm, and so on. The 5S-HM, contains items evaluating putting oneself in harms’ way indirectly that are consistent with indirect self-harm formulated by Green et al. (21).

Direct forms of putting oneself in harms’ way measured by the 5S-HM include swallowing sharp objects (e.g., forks and knives) or ingesting abrasive materials not meant for human consumption, lighting oneself on fire or driving in a reckless manner for the purpose of increasing the likelihood of bringing harm to oneself (20). We include consideration of indirect and direct self-harm in the 5S-HM as they have been repeatedly reported to the first and last authors of this paper in clinical practice, reportedly serving the same function as other forms of self-harm, with therapy-interfering and life-threatening consequences. Because this form of self-harm has not been adequately described in the existing literature, we added it to the 5S-HM to allow for a better understanding of its prevalence and functions.

Sexual self-harm is a more recent development in terms of self-harm research and as such requires careful consideration. First publications defining sexual self-harm and evaluating the phenomena amongst adolescents in Sweden (23) followed from a report from the Swedish Children’s Welfare Foundation articulating this line of enquiry as a priority (24). Fredlund et al. (23) formulated sexual self-harm as an indirect form of self-harm since its damaging effects are not always readily apparent and it does not always cause tissue damage. These authors aimed to evaluate the frequency of “sex as self-injury” (SASI) in relation to demographic characteristics, sexual experiences and risk-taking, child maltreatment and related trauma, as well as mental health functioning and professional help-seeking. They defined sexual self-harm as “… a sexual behavior in relation to another person in order to self-injure.” (p. 3) (23). Although their findings suggested low base rates of participants having used SASI at least once in their lifetimes, amongst those who did use SASI it appeared to be a considerable problem. Over the past year 58% of those who engaged in SASI reported doing so 1–5 times, with 16.3% engaging in SASI more than 5 times.

Youth engaging in SASI were more likely than youth who did not engage in SASI to come from economically disadvantaged families, to live with only one of their parents, to live alone, with a partner, a sibling, in an institution or foster care and be of a sexual minority orientation (23). Selling sex was reported by 11.3% of youth engaged in SASI, and experience of child sexual abuse was reported by 75.0%. Mental health and emotional difficulties were common amongst youth engaged in SASI, in particular anxiety, depression, posttraumatic stress disorder, dissociation, anger, NSSI, and sexual concerns. The authors concluded that SASI is more common among girls, associated with high rates of NSSI, suicide attempts and other mental health difficulties, often arising as a sequalae to child sexual abuse. The relation of SASI to histories of child sexual abuse were explained by the authors as experiencing the body as “damaged goods” wherein it subsequently becomes grounds for further sexual behaviors engaged in for the purpose of self-harm.

To the best of our knowledge there are no similar studies systematically evaluating sexual self-harm defined as such in adulthood. Although sexual behaviors have been included in studies of “risky behaviors” (25), like Fredlund et al. (23) we view indirect behaviors including sexual behaviors used for the function of harming oneself as self-harm. This distinction in formulation is important for several reasons:

1. People in therapy report using sexual behaviors for the same functions as NSSI and other forms of direct and indirect self-harm, at times substituting one behavior for the other if, for example, NSSI but not sexual self-harm was reported as being targeted in therapy as a behavior to terminate (23).
2. Referring to sexual self-harm behaviors as such should give these behaviors a higher priority in treatments that structure therapy targets with suicide and self-harm given attention first and foremost, such as DBT.
3. People in therapy who self-harm using sexual behaviors may be reluctant to disclose this fact, given the risk of facing judgment, stigma and feeling shame. It is also unlikely that people in therapy are being asked about engaging in sexual self-harm by their clinicians since evidence-based treatments to date may not formulate sexual self-harm as such.

In sum, the abundance of self-harm measures has generated a rich literature, although the lack of consensus in formulating robust definitions and parsimonious measures is a limitation in the state of the current research. In the absence of understanding the phenomenology and classification of behaviors encompassed by all possible forms of self-harm, including indirect self-harm such as sexual self-harming behaviors, existing therapeutic interventions targeting self-harm and suicidal behaviors may be limited.

## Aims

The aims of this study are: (1) To evaluate a new self-harm assessment via empirical analyses, (2) To determine novel contributions from the 5S-HM in understanding the forms and functions of self-harm via reasons given for engaging in each type of behavior within a clinical sample, (3) To test the utility and novel contributions of the Unified Model (20) and 5S-HM by extension in understanding and assessing all forms of self-harm for use in both clinical and research settings.

## Materials and methods

### Participants

Participants were *N* = 199 adults (M_*age*_ = 29.98, SD = 8.41; *n* = 172, 86.4% female, *n* = 22, 11.1% male, *n* = 5, 2.5% other/non-binary gender). Participants’ age ranged from 18 to 54. At the time of study participation participants reported living alone (*n* = 48, 24.6%), in a treatment center (*n* = 31, 15.9%), with partner(s) (*n* = 29, 14.9%), with parents, (*n* = 20, 10.3%) or with friends (*n* = 9, 4.6%). Most participants identified as having “other living situations” (*n* = 58, 29.1%), and four participants did not respond to this question. Most participants did not describe their ethnicity. Of those who did, the majority (*n* = 78, 91.8%) described themselves as European. Other ethnicities were described at the individual (*n* = 1) level, which is omitted to avoid the risk of identification A total of 190 participants reported their highest level of completed education, with *n* = 24 (24.2%) completing grade school, *n* = 94 (49.5%) completing high school, *n* = 35 (18.4%) completing University, and *n* = 15 (7.9%) completing another form of education.

Most participants had a diagnosis of borderline personality disorder (BPD: 58.8%). Further diagnoses included neurodevelopmental disorders (25.1%) such as attention deficit hyperactivity disorder, affective disorders (20.6%), trauma- and stressor related disorders (16.1%), eating disorders (13.1%), and anxiety disorders (10.1%). The majority of participants were individuals participating in an ongoing randomized controlled trial (RCT) testing a crisis management model for self-harming and suicidal individuals were also included (26) (*n* = 112), diagnosed by a psychiatrist using the mini-international neuropsychiatric interview (M.I.N.I.) (27) and *Structured Clinical Interview for DSM IV Axis II Disorders* (SCID II) (28). Diagnoses not assessed by these instruments (such as neurodevelopmental disorders) were also collected based on self-report, verified by medical records. For the remainder of participants, diagnostic status was documented by participant self-report, as meeting diagnostic criteria, or having sufficient difficulties to be offered specialized services was a prerequisite for treatment. For demographic characteristics and diagnoses of participants by participating study site, see Table 1.

**TABLE 1:** Frequencies of demographic characteristics and diagnostics by participating treatment site completing the 5S-HM.

Site	Age	Sex	BPD	PTSD	ANX	BD	DD	ED	NEU-RO
1	27.5 ± 5.07	f: 90.0% m: 10.0% other: 0.0%	55.0%	40.0%	15.0%	30.0%	25.0%	30.0%	35.0%
2	28.2 ± 8.10	f: 90.0% m: 10.0% other: 0.0%	72.5%	32.5%	25.0%	22.5%	27.5%	15.0%	25.0%
3	31.5 ± 6.12	f: 90.9% m: 9.1% other: 0.0%	9.1%	9.1%	9.1%	18.2%	36.4%	54.6%	9.1%
4	31.5 ± 9.09	f: 83.0% m: 12.5% other: 4.5%	58.0%	2.7%	0.9%	0.0%	0.9.%	3.6%	21.4%
Supplemental site 1	25.8 ± 6.18	f: 93.8% m: 6.3% other: 0.0%	68.8%	43.8%	31.2%	0.0%	3.0%	25.0%	50.0%

Site 1, specialized residential treatment site; site 2, specialized outpatient treatment; site 3, eating disorder center; site 4, RCT participants, Supplemental site 1, supplementary assessments from site 1. f, female; m, male; BPD, borderline personality disorder; PTSD, trauma/stress- related disorder; ANX, anxiety disorders; BD, bipolar and related disorders; DD, depressive disorder; ED, eating disorder; Neuro, neurodevelopmental disorders. Diagnoses with frequencies of < 5% are not documented.

This was a multi-site study of patients from specialized settings, using two different data collection methods: interview and online self-ratings. The samples were convenience samples, with participating research sites joining the study to contribute to the development of a novel and comprehensive self-harm assessment, the 5S-HM. For this reason, an eating disorder site participated alongside sites offering evidence-based treatment and novel interventions for individuals with BPD and pervasive suicidality.

Participants were recruited for the 5S-HM study from four different sites: a residential treatment center with DBT as its primary therapeutic model (site 1: completed *n* = 20 5S-HM interviews). Individuals from a general psychiatric ward, and from psychiatric day treatment and the DBT team of an adult psychiatric hospital (site 2: *n* = 40 completed 5S-HM interviews). Sites 1 and 2 were the only sites whose participants completed the 5S-HM in interview format. Individuals from a regional eating disorder center (site 3: completed *n* = 11 online versions of the 5S-HM assessment package). Participants from two additional sites completed self-assessments with 5S-HM online: individuals in the aforementioned RCT (site 4: completed *n* = 112 online assessments) alongside a second cohort of residentially DBT-treated participants using the online version of the 5S-HM (supplementary assessments from site 1: completed *n* = 16 online assessments). In sites 1–3 the 5S-HM was assessed twice with an interval of 14 days after the first assessment (*n* = 49 T2 assessments).

#### Inclusion and exclusion criteria

Inclusion criteria were adult age (18-years-old and older), being a current recipient of mental health services, willingness, and ability to give informed consent and having self-harmed or attempted suicide on two or more occasions over the past six months. The singular exclusion criterion was intellectual disability (ID) given that it may take abstract reasoning skills to reflect and describe one’s emotions and to formulate reasons for self-harming behaviors. While we do believe that reflecting and describing emotions and reasons for behaviors are possible amongst individuals with ID with support, we lacked the resources to work with them in doing so.

### Measures

#### Inventory of statements about self-injury (ISAS)

The ISAS (7) is a self-assessment questionnaire, which queries frequencies of different types of NSSI as well as descriptive characteristics that the respondent is asked to consider in relation to their behavior. The ISAS consists of statements aimed at identifying the relative importance of different possible functions of NSSI. There are three response options ranging from “*0 = not relevant*,” to “*2 = very relevant*.” There are two overall categories in the ISAS: intrapersonal functions and interpersonal functions. A psychometric study of the ISAS (7) has shown high internal consistency with Cronbach’s alphas of 0.88 for the interpersonal scale and 0.80 for the intrapersonal scale. The Swedish version of this measure (29) was used in the present study.

#### The difficulties in emotion regulation scale (DERS)

The DERS (30, 31) is a self-report measure that examines participants’ ability to identify, understand, accept and modulate emotions as well as respond effectively to the environment despite one’s emotional state. The DERS has six subscales. These are lack of emotional awareness, lack of emotional clarity, impulse control difficulties, difficulties engaging in goal-directed behavior, non-acceptance of emotional responses, and limited access to emotion regulation strategies. In this study, only the total scale was used. Response options are on a Likert-type scale ranging from “*1 = almost never*” to “*5 = almost always*.” The DERS has good psychometric properties with good construct, predictive and test-retest reliability as well as high internal consistency (30, 32). Initially a Swedish 36-item version of the DERS was used in this study (30). However, over the course of data collection, the 16-item version of the DERS was validated in Swedish (31), which we used for data collection in this study from 2016 until the completion of data collection in 2018. At study completion we unified our datasets by extracting the 16 items from the short-form of the DERS-16 which was used for final analyses in the study. The 16 items were evaluated prior to unification of the dataset to ensure that the content of each item was the same.

#### Demographic background questions

This set of questions queries participants’ age, sex, gender identity, marital status, current living arrangement and educational achievement. Diagnostic status is also queried.

#### The five self-harm behavior groupings measure

This is an extensive measure of self-harm based upon a review of the literature and clinical experience working with this population. It was generated from the Unified Model to more accurately measure behavior across five self-harm behavior groupings, across two spectra: from low to high directness and low to high lethality (20). Supplementary File 2 lists the five groups of behaviors measured by the 5S-HM, with a sample item from each behavior group.

Participants were asked whether they engaged in each of the 35 5S-HM behaviors over the past 2 weeks (Y/N), followed with the statement: “*If yes, how many times over the last two weeks?*” response options included four categories: “*One time, 2–3 times, 4–5 times, 6 or more times*.” These response options are weighted so that more frequent and more severe behaviors have a higher score. For example, going one time without adequate food or adequate nutrition (one of the least severe items) would be scored as 1, while a suicide attempt would be scored as an 8 if it happened one time during the last 2 weeks (details regarding the weighting are provided in the 5S-HM in Supplementary File 1). The 35 behaviors from the 5S-HM generate a weighted total score ranging from 0 (i.e., none of these behaviors has occurred) to 400 (i.e., all the 35 behaviors have been engaged in 6 or more times during the last 2 weeks). At the conclusion of each section of the 5S-HM the participant is asked if there was anything else they did to harm themselves in a similar way over the past 2 weeks. If so, that behavior is documented. Suicidal intent is also queried in relation to each 5S-HM section. The 5S-HM takes between 30 and 45 min to administer by interview, and approximately 15 min to complete in its online version. It is available in English and Swedish.

#### Reasons for engaging in self-harm

Following each 5S-HM section participants were asked their three most important reasons for engaging in the self-harm that they endorsed in the previous section. They are also asked to rank their reasons for engaging in self-harm in order from most to least important.

The words and phrasing of questions in the 5S-HM and demographic background questions have been reviewed by representatives from the Swedish regional LGBTQIA + organization (Riksförbundet för sexuellt likaberättigande: RFSL), and from a group of individuals with lived experience or interest in self-harm and eating disorders (Self-Harm and Eating Disorders Organization: SHEDO). A sex worker was asked for their feedback regarding the neutral phrasing of the sexual self-harm behaviors. All feedback in relation to avoiding stigmatizing and pathologizing language was followed as exactly as possible.

### Analyses

#### Empirical analysis

The 5S-HM provides a detailed assessment of frequency, directness, and lethality of self-harming behaviors across five domains both on an item level and on an aggregate level. In addition, the 5S-HM generates a total score described above. The total score is designed as a quick reference for overall level of current self-harming and suicidal behaviors, which provides an index for comparing repeated assessments over time. Internal consistency of the 5S-HM total score was established from Cronbach’s alpha. Test-retest reliability was evaluated by calculating intraclass correlations (ICC) to the repeated measurements of the 5S-HM total score. Construct validity of the 5S-HM total score was tested by correlating the 5S-HM total score with conceptually convergent scores from established assessment instrument: (i) the total number of NSSI behaviors as assessed with the ISAS (28), (ii) the DERS (31) total score (short version). Because some of these scales were highly skewed, particularly the total number of NSSI behaviors from the ISAS, correlations were based on ranks (Spearman correlations).

#### Thematic analysis

Inductive TA was used to describe data by examining the reasons given for engaging in each type of self-harming behavior (33). Ranked reasons for engaging in self-harm behaviors were reviewed to determine whether there was variance in the first, second, and third-ranked most important reason reported by participants. Participants tended to give the first-ranked reason the most detail, and then have some repetition or leave blank the second and third reasons for engaging in a specific form of self-harming behavior. Even within the first-ranked reason given for self-harming behavior there were a number of responses that were incomplete or were not clearly tied to the question. To conduct TA using as much relevant data as possible, rather than trying to use all the data regardless of content (34) we present only the first-ranked reason for engaging in each type of self-harm at T1 interview, and only reasons endorsed by 10% of the sample or more.

This qualitative approach is a data driven process wherein codes and themes are developed by summarizing descriptive verbal responses rather than trying to fit them into a pre-existing coding scheme (33, 34). This approach was followed insofar as not interpreting data and generating themes that mapped as closely to the actual data as possible (that is, verbatim).

### Procedure

Participants were sequentially recruited at each setting by a study coordinator already working within each setting, as a scientist-practitioner or therapist. Study coordinators were responsible for overseeing the assignment of study code numbers that were used to identify participants, as well as to ensure that Time 1 and Time 2 surveys were completed 14 days apart (sites 1–3) and to assign links for electronic data entry in sites 3 and 4, and supplementary online assessments at site 1. Study coordinators met the principal investigator (PI) monthly to update participant lists and to transfer interview data for data entry and confidential storage (sites 1 and 2). The PI also communicated with the study coordinator at study site 3 monthly to update study IDs. Electronic data generated by sites 3 and 4 and supplementary online assessments at site 1 were managed and housed within Sunet Survey, a commercial IT system procured in accordance with the Swedish Public Procurement Act, which is Lund University’s encrypted web-based server for data entry and retrieval.

At site 1 and 2 therapists invited individuals to participate if they met inclusion criteria. Therapists also completed the consent procedure and participant interviews. At sites 3 and 4 the study coordinators were scientist-practitioners who oversaw the consent procedures and confidential storage of study materials and assignment of links to Sunet Survey. Online assessments completed at supplementary site 1 followed a mixed procedure whereby participants were recruited by study coordinators and had their consent procedures managed by their therapist who also gave them links for electronic study participation. Participants at site 3 were given a link to complete assessments on their own, whereas participants at site 4 and supplementary online site 1 were in hospital-based or residential care at the time of study participation.

For sites 1 and 2 using the interview version of the 5S-HM, study coordinators and therapists recruiting participants were given a one-hour training session to guarantee a uniform administration of the interview. A one-hour training session on managing self-harm and suicide more generally was given (as requested) to the eating disorder site (3) along with a 30-minute training regarding procedures for assigning code-numbers and assigning electronic links for study participants. The 30-minute training on procedures for electronic study participation was also given to site 4. Training on consent procedures for all staff was delivered to all participating sites.

#### Thematic analyses

Interview data were de-identified, translated from Swedish to English and analyzed using six phases of TA outlined by Braun and Clarke (33). The entire dataset was reviewed for familiarization (Step 1) by the first author with codes generated for any reason that occurred in the dataset on two or more occasions (Step 2). Reasons that occurred only once were coded in an “other” category. This process initially generated 79 codes of reasons. These codes were reviewed by authors 1, 2, 4 and 5, and gathered under overarching themes and subthemes created from the content of the codes (Step 3). One principle applied in this process was to differentiate between codes for reasons preceding the act of self-harm behavior “antecedents” and codes for reasons following the act of self-harm “consequences.” For example, negative emotional states preceding the act of self-harm was a separate theme from anxiety reduction following the act. The fourth author then generated a preliminary thematic map (Step 4), depicting the themes and subthemes over time that was refined by the first author (Step 5) to confirm the final themes as well as the relationships between themes and subthemes underlying the data. Step 6, the final analysis, is based upon 5 themes and 13 subthemes depicted the thematic map (please see Figure 2) describing self-harm over time as expressed by study participants in the 5S-HM.

All study procedures and measures were approved by Lund’s Ethics Examination Board (Diary Number: 2015/517 and 2014/570 for the RCT participants).

## Results

### Results of empirical analysis

As illustrated in in Table 2 the most prevalent form of self-injury was harmful self-neglect (Group 2 behaviors). Group 2 behaviors must be interpreted with caution, since they query items such as deliberately not eating or drinking enough for the purpose of self-harming. However, many participants endorsed not eating or drinking enough and gave the reason that they did so to diet or due to lack of money for groceries. Their reasons were dropped from qualitative analysis since they were not engaged in these behaviors for the purpose of self-harming. Direct forms of self-injury from Group 1 such as cutting with a razor, knife, scissors, broken glass, or other implement were highly prevalent with 66.7% of the participants engaging in at least one form of direct NSSI over the past 2 weeks (for details see Table 2).

**TABLE 2:** Frequencies of specific forms of self-harm behaviors during the last 2 weeks.

5S-HM behavior group and items	% of participants engaging in this behavior during the last 2 weeks
**1. Self-injury (direct), 66.7%**	
1. Scratching yourself with nails or another tool (including biting yourself)	32.1%
2. Carving or puncturing your skin	22.8%
3. Cutting yourself with a razor, knife, a pair of scissors or broken glass	27.3%
4. Rub anything corrosive into your skin or specific body-part (like household cleaner)	0.5%
5. Burned yourself	9.6%
6. Interfere with the healing or wounds on your skin	27.9%
7. Other similar behaviors used for the same purpose?	24.1%
8. Banging your head against a hard surface	25.3%
9. Banging your arms, legs, hands, feet, or sides against a hard surface to cause a bruise or break a bone?	19.0%
10. Deliberate injury of any other body part	12.4%
**2. Harmful self-neglect (indirect), 75.4%**	
11. Deliberately not taking medicine that was prescribed to you (not including forgetting)	22.8%
12. Not seeing a doctor for a new or chronic health condition	13.1%
13. Deliberately going without the sleep you needed for more than three nights in a row	15.2%
14. Misuse of prescription or over-the-counter medication	31.1%
15. Deliberately gone without adequate food or nutrition	61.6%
16. Deliberately gone without hydrating fluid	19.9%
**3. Sexual self-harm/exploitation (indirect), 20.2%**	
17. Deliberately having sex despite not wanting to and with the skills/ability to say no	13.7%
18. Having multiple sex partners on one occasion as an expression of self-harm	1.2%
19. Having unprotected sex with stranger(s) without asking about sexual health	10.2%
20. Have unprotected sex with partner(s) known to have sexually transmitted infections for the purpose of contracting the infections?	0.0%
21. Was there any other way you used sexual behaviors (even indirectly for example online or through communication by mobile phone) to harm yourself over the last 2 weeks?	8.3%
**4a. Putting oneself in harms’ way (indirect), 40.8%**	
22. Walking into a busy street without checking traffic for oncoming vehicles	30.1%
23. Picking or provoking an extremely uneven physical fight to put yourself in harms’ way	2.1%
24. Walking alone in neighborhoods known to be unsafe due to street violence as a method of exposing yourself to risk	8.7%
25. Using substances known to be associated with extremely adverse outcomes to put yourself in harms’ way?	4.6%
26. Contacting or involving yourself in organized crime to put yourself in harms’ way	0.5%
27. Other behaviors used to put yourself in harms’ way	12.9%
**4b. Putting oneself in harms’ way (direct), 18.8%**	
28. Swallowing non-ingestible objects or substances	2.5%
29. Trying to choke, strangle or hang yourself	10.2%
30. Engaging in extreme risk-taking? E.g., trying to light yourself on fire	4.6%
31. Firing a weapon at your body for the possible risk of killing yourself	0.0%
32. Driving in an extremely reckless manner	4.1%
33. Standing, lying, or jumping onto train tracks when there was a train approaching	2.8%
34. Other way to put yourself directly in harms’ way?	14.8%
**5. Suicide attempt (direct), 17.1%**	
35. Attempt to end your life?	17.1%

The 5S-HM further revealed that 20.2% of the participants engaged in at least one form of sexual self-harming behavior during the lasts 2 weeks including unwanted sex for the purpose of self-harming (13.7%) and unprotected sex with strangers for the purpose of harming oneself (10.2%). Further self-harming sexual behaviors such as having sex with multiple partners for the purpose of self-harm (1.2%) were relatively rare. The importance of assessing self-harm behavior beyond classical forms of NSSI or suicide attempt is further illustrated by the incidence of section 4A and 4B behaviors, which refer to putting oneself in harms’ way indirectly and directly, respectively.

As many as 17.1% of the participants reported that they had attempted to end their lives over the past 2 weeks. The majority of these participants’ 5S-HM data are derived from baseline assessments from participants beginning an RCT to test the efficacy of a novel crisis management model for pervasively self-harming and suicidal individuals, for whom recent hospitalization had been necessary (26). To evaluate similarities and differences in the sample with respect to suicide attempts outside of the RCT participants, the prevalence of suicide attempts over the past 2 weeks based on the 5S-HM from participants other than those in the RCT subsample was calculated. Results indicated the same frequency as analyses with the RCT participants: 17% (SD = 0.38). This may reflect the severity of difficulties experienced by those completing residential treatment, who have almost always tried less intensive therapies with limited therapeutic gains prior to residential treatment.

**FIGURE 1:**
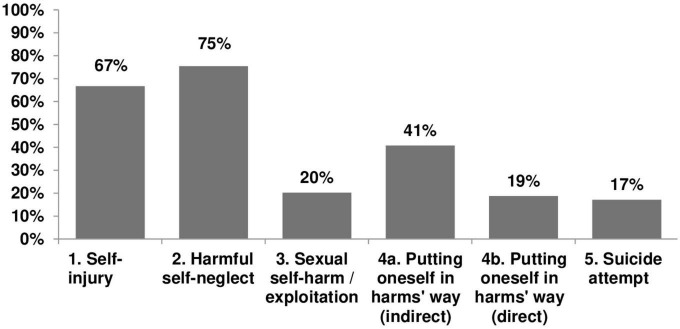
Rates of self-harm measured by the 5S-HM over the past 2 weeks.

The rates of self-harming behaviors reported by participants over the past 2 weeks are depicted in Figure 1 and summarized in Table 2.

**FIGURE 2:**
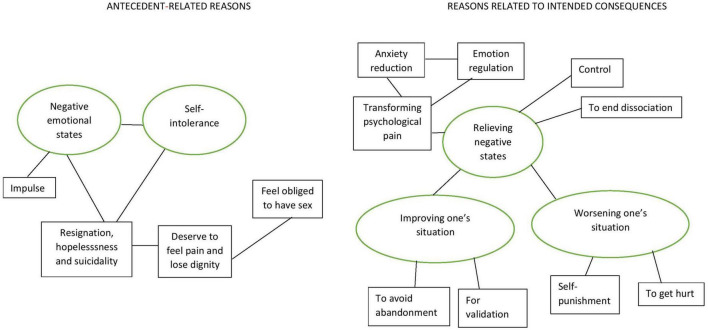
Thematic map depicting antecedents and consequences of self-harm behavior derived from the 5S-HM. A general distinction was made between themes reflecting reasons related to experiences preceding the self-harming behavior and themes reflecting reasons related to intended consequences of the self-harming behavior. The first two themes (from left to right) belonged to the first category: (1) negative emotional states and (2) self-intolerance. The three remaining themes belonged to the second category: (3) relieving negative states, (4) improving one’s situation, and (5) worsening one’s situation. The relationships between main themes (in ellipses) and subthemes (in squares) are illustrated by lines connecting them.

Cronbach’s alpha of the 5S-HM total score was 0.75 indicating acceptable to good internal consistency according to widely accepted standards. The test-retest reliability is supported by an intraclass correlation of 0.68 considered good [e.g., according to the guideline by Cicchetti (35)]. The construct validity of the 5S-HM is supported by significant positive correlations between the 5S-HM total score and (i) the total number of NSSI behaviors as assessed with the ISAS (28) (rho = 0.40, *p* < 0.01), (ii) the DERS (31) total score (rho = 0.26, *p* < 0.01).

### Results of thematic analysis

As described earlier, five main themes and 13 subthemes (please see Table 3) were developed through TA of the primary reasons given by the participants for engaging in the different types of self-harming behavior. A general distinction was made between themes reflecting reasons related to experiences preceding the self-harming behavior and themes reflecting reasons related to intended consequences of the self-harming behavior (please see Figure 2).

**TABLE 3:** Main themes and subthemes generated from first-ranked reasons for engaging in self-harming behavior in the 5S-HM grouped by antecedents and intended consequences.

Reasons related to	Main themes	Sub-themes
Antecedents	1. Negative emotional states	1. Impulse 2. Resignation, hopelessness, and suicidality
	2. Self-intolerance	2. Resignation, hopelessness, and suicidality 3. Deserve to feel pain and lose dignity 4. Feel obliged to have sex
Intended consequences	3. Relieving negative states	5. Anxiety reduction 6. Emotion regulation 7. Transforming psychological pain 8. Control 9. To end dissociation
	4. Improving one’s situation	10. To avoid abandonment 11. For validation
	5. Worsening one’s situation	12. Self-punishment 13. To get hurt

#### Theme 1: negative emotional states

This theme reflects the substantial role of overwhelming negative emotions and states driving self-harm behavior. Discrete negative emotions given as reasons for engaging in self-harming behavior were loneliness, sadness, feeling stress, distressed, frustrated, desperate, angry, sad, and empty. “*Wanting to disappear*” was also a main reason for self-harming endorsed by several participants who were motivated by negative emotional states. Broader negative emotional states such as tension were very often described as emotional experiences that built over several hours or days. As such they were described as overwhelming, unbearable, and inescapable. Subthemes of Impulse and Resignation, hopelessness and suicidality are encompassed within this first main theme.

### Impulse

This subtheme described how a proportion of participants habitually engaged in self-harm to the extent that they now used the behavior out of impulse rather than due to thinking through their situation, including other possible alternatives. “*Not thinking about consequences*” was a succinct quote from a participant. This subtheme falls within the overarching theme of negative emotional states because it was most often that participants reported self-harming on impulse once they were already feeling stuck within experiences of overwhelming negative emotion. In sum, self-harming on impulse when feeling badly was a meaningful reason given for self-harming by many participants.

### Resignation, hopelessness, and suicidality

This subtheme was encompassed by both the main theme of negative emotional states and the main theme self-intolerance (see below). The primary semantic content of this subtheme was related to suicidal thoughts and related experiences such as “*feeling tired of life*” or “*tired of illness*”; “*feeling hopeless*” and “*as though* [I] *cannot live*”; being tired of mental illness and living with trauma; being unwell and feeling that there is nothing to live for. Participants described feeling no meaning in life and inability to tolerate living as well as a sense of “*already feel*[ing] *dead.*” Taken together, these experiences contributed an important aspect of reasons given for engaging in self-harm for participants within the study.

#### Theme 2: self-intolerance

This main theme encompassed participants’ overwhelming and often self-defining experiences of themselves as hateful in relation to primary reasons given for self-harm. Participants often described self-harming due to their experiences of their own self-hatred, self-disgust, “[sense of] *being contaminated*”, and “*worthlessness*.” Participants also described body disgust, contempt and hatred as associated with the experience of self-intolerance. Subthemes of resignation, hopelessness, and suicidality, deservingness to feel pain and lose dignity, and feel obliged to have sex are encompassed by the self-intolerance main theme (see Table 3 and Figure 2).

### Deserve to feel pain and lose dignity

This subtheme was comprised by participants’ experiences reporting that they self-harmed due to “*not feeling good or worthy enough to have a good life*.” Similar reasons for self-harm within this subtheme was the experience of “*not feeling worthy of having body integrity*” or not feeling worthy of “*saying no*.” This subtheme was related to further antecedent subthemes of feel obliged to have sex, as well as the subtheme of resignation, hopelessness, and suicidality.

### Feel obliged to have sex

This subtheme related to participants’ sense that they needed “*to obey*,” or “*not disappoint someone*” else in sexual contexts. Participants who reported this experience often also described “*feelings of guilt*,” or “*wishing to please*” to the exclusion of their own sexual desire or interest. One participant summarized her experience of feeling obliged to have sex and “*not daring to say no*.” Lack of self-worth in the context of feeling obliged to have sex was described by another participant, who stated that “*sex* [is the] *only thing I am good for*.” This sentiment was echoed by other participants who described having unwanted sexual experiences as a form of self-harm.

#### Theme 3: relieving negative states

This main theme is the first of three main themes reflecting reasons related to intended consequences of self-harming behavior (see Table 3 and Figure 2). Relieving negative states encompassed participants’ expectations of what would follow from engaging in one or more behaviors listed in the 5S-HM. Main theme #3 was further linked to subthemes Anxiety reduction and Emotion regulation which were further associated with the subtheme Transforming psychological pain, subtheme of Control and the subtheme To end dissociation. Main theme #3 was further linked to main theme #4 Improving one’s situation and main theme #5 Worsening one’s situation.

The essence of main theme #3 was related to participant experiences of self-harm generating a “*change* [of] *mental or emotional state*.” Self-harm was used to break the tension, anxiety and other negative emotions and experiences that were perceived to be overwhelming and unbearable as described in main theme #1 which initiated a self-harm behavioral sequence.

### Anxiety reduction

This subtheme was related to participants’ experience of how self-harm behavior reduced their anxiety in general and specific ways. Participants described feeling reductions in not only anxiety, but also reductions in tension as well as ending flashbacks through self-harm. As one participant described: “*the pain itself is anxiety reducing*.” This subtheme was linked with the subthemes of emotion regulation and transforming psychological pain as follows.

### Emotion regulation

Within the subtheme of Emotion regulation were participant experiences of using self-harm for the purpose of “*distraction*,” “*avoidance*” and to “*manage*,” “*express*,” “*terminate*” or regulate negative emotions or “*escape the present*.” Participants further described self-harming for the purpose of calming and soothing oneself, and “*numbing myself*…*to black out*.” These were many participants’ main reason for self-harm.

### Transforming psychological pain

This subtheme related to participants’ description of how the experience of self-harm helped to release mental, “*psychic*” or spiritual pain. One participant stated that through self-harm she could “*transform suffering into physical pain*” which was perceived to be less chaotic and unmanageable than the emotional pain.

### Control

This subtheme of Control was described in relation to self-harm whereby self-harm was used for this function in relation to emotions, oneself, or one’s environment. Control was described as desirable because it gave a sense of autonomy when other aspects of life were perceived as being overly controlled by others or out of the control of the individual. Control was also described in relation to suicide “*I want to control my own death*”; “*I want to control how I die*.” Taken together, self-harm for the purpose of or in relation to control was a unique reason some participants reported using self-harm behaviors.

### To end dissociation

This subtheme was comprised of participant experiences whereby self-harm was used to end dissociation and related symptoms and unwanted experiences. Participants described using self-harm to end or break experiences of numbness, “*to feel real; to feel I exist*,” as well as to prevent or terminate dissociation once they felt its onset.

#### Theme #4: improving one’s situation

Many participants’ main reasons for self-harming were related to a desire to improve their situation or make things better for themselves through self-harm. This was sometimes in relation to care and healthcare, while on other occasions it arose in the context of personal relationships. This main theme encompasses subthemes To avoid abandonment and For validation as follows.

### To avoid abandonment

This subtheme was expressed in care-giving contexts, in social relationships and in sexual contexts. In caregiving contexts some participants described self-harming in order to receive “*care-taking*.” Relatedly some participants described self-harming due to a “*fear of being well*” and losing care for that reason. Socially, some participants described using self-harm to “*keep company*”; “*be thought of*” or “*not be left* [alone].” One participant described self-harm and specifically sexual self-harm to try to “*guarantee* [a] *relationship*” and “*avoid abandonment*.” That is, it was hoped that a relationship would be guaranteed if the participant allowed her partner to hurt her.

### For validation

This subtheme was also associated with Theme #4 of using self-harm in an effort to improve one’s situation. Feeling liked or desired was described by participants using self-harm for this purpose, most often in the form of sexual self-harm. Accordingly, it was a unique contribution to understanding their main reason for self-harm.

#### Theme #5: worsening one’s situation

The final consequential main theme in our analyses related to using self-harm as a way of increasing pain and suffering. The subtheme of Self-punishment, and To get hurt were encompassed by this main theme, which had at its core a sense of deservingness to ultimately suffer by experiencing more multifaceted pain.

### Self-punishment

This subtheme was comprised by participant experiences in which participants described self-harm engaged in for the purpose of demonstrating worthlessness: “*to make* [myself] *ugly*”; “*so that it will hurt*” and to demonstrate that they were “*not worthy of help*.” Self-harm for the purpose of self-punishment was frequently endorsed as a main reason for self-harming.

### To get hurt

The final subtheme in our analyses that was encompassed by main theme #5 had to do with a sense of deservingness to suffer in relation to sexual self-harm along with the intention for sexual self-harm to be “*more painful than other forms of self-harm*.” This subtheme was comprised of reasons given for self-harming by participants such a “*wanting to be sick*”; “*wanting to hurt*”; “*wanting to feel pain*”; to “*show illness*.” Some participants described choosing indirect self-harm to avoid detection: “*I hoped to get hurt without getting into trouble for it*.”

Taken together alongside the patterned nature of self-harming behavior, it may be that participants moved through experiences of negative emotional states and self-intolerance to self-harm behavior, from which they expected relief from painful states or improvement in their relations to others, or an even worse suffering they felt deserving of. This TA might suggest a model in which participants find themselves in experiential loops of antecedents leading to self-harm which in turn leads to consequences that pave the way for further antecedents and repeated self-harm.

## Discussion

We aimed to evaluate a new self-harm assessment through empirical analysis and to determine the novel contributions from the 5S-HM in understanding the forms and functions of self-harm via qualitative analysis of reasons given for using each type of self-harm behavior described. We also aimed to test the utility and novel contributions of the Unified Model (20) and 5S-HM by extension in understanding and assessing all forms of self-harm for use in both clinical and research settings. In this study we generated initial analyses regarding internal consistency, test-retest reliability, and construct validity, which indicate that the 5S-HM is suitable for the assessment of a spectrum of behaviors encompassed by self-harm.

In interpreting the 5S-HM, the total score generates a comprehensive index to evaluate self-harm. It is important to note that the 5S-HM total score does not suggest that NSSI and suicidal behaviors are the same thing phenomenologically or otherwise, but rather are end-points on a self-harming spectrum (20, 36–38). Behaviors are weighted by lethality so that life-threatening behaviors in Group 5 are weighted more heavily than NSSI behaviors in Group 1. For this reason, behavior groupings should also not be compared to each other to interpret magnitude or clinical severity. To understand the role of self-harm as measured by the 5S-HM, results of each individual behavior grouping must be examined alongside the reasons the person gave for self-harming in that form, as well as whether or not the behavior was associated with suicidal intent.

Engaging in self-harm measured by each behavior grouping evaluated by the 5S-HM in our clinical sample revealed that direct self-harming behaviors such as NSSI were common, engaged in for some of the same reasons as reported by other evaluations of NSSI in non-clinical samples such as for tension reduction and affect regulation (7, 39). Affect dysregulation and self-harm have demonstrated a positive association in community samples (40). Improving affect regulation capacity is associated with reduction in self-harm behaviors (41). Accordingly, our findings support good convergent validity between the 5S-HM and the difficulty with emotion regulation scale (DERS) (31).

Our results indicate that indirect forms of self-harm, such as harmful self-neglect are also common. This suggests that mapping the entire self-harming repertoire may be critical for accurate case formulation and treatment, as much of an individual’s experience may be shaped by, for example, chronically under-sleeping, deliberately not drinking enough fluid in order to induce dissociation, allowing treatable conditions to become chronic through lack of intervention, and so on. In fact, not tending to these concerns may contribute to lack of clinical improvement. In sum, we believe that clinical and research assessment of harmful self-neglect is a vital and under-studied area.

Novel findings from this study are also present in the rates of indirect (41%) and direct (19%) self-harm related to putting oneself in harms’ way (5S-HM behavior groupings #4a and #4b). A further novel finding was that one fifth of participants reported engaging in sexual self-harm over the previous 2 weeks.

Thematic analyses indicated that reasons for self-harming sometimes were expressed in terms of intolerable emotional states leading up to self-harming, sometimes in terms of consequences awaited to follow self-harming. Participants described negative emotional states and self-intolerance as culminations of suffering and self-hatred, respectively. The weariness of being ill and the habit of self-harm led to self-harming behavior for some, as did the sense of deservingness to feel pain and lose dignity, particularly in relation to feeling obliged to have sex and engaging in sexual self-harm for others. Participants also described relief from negative states as the purpose of self-harm, as well as intentions to either improving one’s situation or worsening one’s situation through self-harm. Being motivated by relief from negative states is a relatively well-documented function of self-harm (7, 39), as is the intrapersonal function of attempting to improve one’s situation (intrapersonal reasons) (42). However, using self-harm to change one’s situation for the worse, particularly in relation to sexual self-harm is a novel contribution of the current study. We interpret this finding as a way of expressing one’s self-hatred rather than masochism, as there was no element of enjoyment or gratification described by our participants who engaged in sexual self-harm.

Findings from thematic analyses in relation to consequences of sexual self-harm indicated that with respect to improving one’s situation, participants engaged in sexual self-harm to try to “guarantee” relationships with people they allowed to hurt them at best or at least to avoid being abandoned. Other participants described using sexual self-harm to feel desired, affirmed or validated. With respect to worsening one’s situation participants engaged in sexual self-harm to express their self-hatred by allowing someone else to hurt them, in some instances with awareness that the pain would be greater than what they had the capability to inflict on themselves. Sexual self-harm was also engaged in for the purpose of being hurt in a way that would avoid external detection. These findings generate insight into how sexual self-harm may be initiated and maintained from an interpersonal/transactional perspective. In other words, it seems likely that others have reinforced beliefs maintaining these behaviors, possibly in the form of earlier sexual trauma (23).

There were limitations to this study. Our convenience sample was comprised of data from multiple treatment settings for self-harming or eating disordered individuals, who were not diagnostically homogeneous. However, a diverse population may be beneficial with respect to ecological validity, as many people receiving psychiatric services self-harm (2, 3). A large sample was required to empirically test the 5S-HM, which is a moderately lengthy measure, making the online version, which is easier to complete quickly, desirable. However, methodological issues arise with respect to using both interview and online measures, the latter of which may have limited our qualitative findings. Time constraints and participant burden prevented in-depth and nuanced querying of responses in the sections generating reasons for self-harm. Accordingly, the qualitative data that were collected were short statements, sometimes consisting of only one word or a sentence.

A further limitation of the study is that our participants were relatively young and predominantly female. While our sample may reflect the most commonly presenting demographics of people who self-harm (that is, young women or female-identifying people), it would be valuable to evaluate the 5S-HM amongst men and male-identifying people as well as individuals who identify as non-binary or elsewhere on the gender spectrum. Understanding self-harm using the 5S-HM in a more diverse sample with respect to biological sex and gender identity could help to advance the field of self-harm research.

Although we believe that that 5S-HM is suitable for assessing self-harm in adolescents, our sample was restricted to participants aged 18 or older. Accordingly, our findings may not generalize to the experience of youth. Given the well-documented negative consequences of sexual abuse during childhood and adolescence as well as the high prevalence of sexual self-harm in our sample, further research is needed to understand the prevalence and reasons given for engaging in sexual self-harm in youth.

Further qualitative study is needed to clearly differentiate between sexual self-harm and sexual abuse. Within the 5S-HM (Supplementary File, p. 10) we define sexual self-harm as “*Engaging in sexual activity without interest, curiosity, or lust, but rather for the purpose of harming yourself. That is, deliberately having sex despite not wanting to and with the skills/ability to say no to a partner you feel confident would have stopped without consequence if you had said no*.” Despite this definition and qualification, some reasons reported for engaging in sexual self-harm, such as “feeling obliged to have sex” and “not daring to say no” may be formulated as sexual abuse in the absence of further context regarding the situation that participants are describing. The 5S-HM would lend itself to in-depth interviews including timelines related to sexual self-harming behavior, to better determine whether and how agreements and contingencies such as consent and abuse, punishment or exploitation arise and possibly change over the course of the event.

How the consequences of sexual self-harm are experienced as reasons for continuing these behaviors is consistent with how much some participants in our study reported feeling one-dimensional or unreal, hating themselves to the point of experiencing self-intolerance, feeling disgusted with their bodies, feeling deserving of pain and loss of dignity. It is also understandable that such overwhelming and unbearable negative experiences and emotions set the stage for a need for further escape, reinforcing the use of self-harm to manage or cope in the absence of other skills or strategies. Further research is required to determine how these variables fit together toward targeting sexual self-harm in treatment.

With respect to future research and treatment development, the vulnerability to exploitation emerging from the reasons participants gave for engaging in sexual self-harm suggests consideration for the development of specific skills training interventions. A format beginning with psychoeducation regarding fundamental rights in relation to having one’s dignity and body integrity respected in public and private appears warranted. Skills training focused upon learning to value oneself outside of sexual arenas could help to undo the formulation of the self as only valuable for sexual use by others. “*Deservingness to suffer*” also warrants future study, as does the subjective experience of pain amongst self-harming individuals (43). This paper presents thematic and empirical analyses illustrating the novel aspects and robustness of the 5S-HM for use in research and clinical settings. Future research will explore if a brief version of the 5S-HM will yield consistent data as the 5S-HM in its current format.

## Data availability statement

The raw data supporting the conclusions of this article will be made available by the authors, without undue reservation. Qualitative data will be reviewed to ensure de-identification prior to being made available.

## Ethics statement

The studies involving human participants were reviewed and approved by the Regional Ethics Board Affiliated with Lund University and Lund University Hospital in Lund, Sweden, which was the primary center in which the study was conducted (Etikprövningsmyndigheten: EPM). Examination Board (Diary Number: 2015/517). Diary Number: 2014/570 allowed us to use the 5S-HM for data collection and secondary analysis from the Brief Admission Skåne participants. An ethics amendment was sought and granted (Diary Number: 2017/644) to allow Psychiatry Residents and senior students completing their Masters’ degrees in the Psychologists’ Program at Lund University to collect data for their theses using the 5S-HM battery. Their interviews contributed data to the sample used in the current study. The patients/participants provided their written informed consent to participate in this study.

## Author contributions

SIL conceived of the study. SIL created the 5S-HM in consultation with SW. SIL and SW collected the data which was electronically managed and stored by DD. SIL wrote the manuscript with feedback from SW, MW-L, DD, and NK. NK completed quantitative analyses and wrote the section on quantitative analyses. SIL, SW, DD, and MW-L completed the steps 1, 2, and 3 of thematic analysis (TA). SIL completed steps 4, 5, and 6 of TA in consultation with MW-L. MW-L generated the initial thematic map that SIL refined. All authors contributed to the article and approved the submitted version.
