# Supplementary material for: The five self-harm behavior groupings measure: empirical and thematic data from a novel comprehensive self-harm assessment
Source: Front Psychiatry. 2023 May 5;14:1147206. doi: 10.3389/fpsyt.2023.1147206 (PMC10196393; doi:10.3389/fpsyt.2023.1147206)
Supplement: Supplementary file 1 [file Data_Sheet_1.docx]

Supplementary Material

**The Five Self-Harm Behavior Groupings Measure: Empirical and thematic data from a novel comprehensive self-harm assessment**

Sophie I. Liljedahl^1, 2, 3^, Daiva Daukantaité^4^, Nikolaus Kleindienst^5^, Margit Wångby-Lundh^4^, Sofie Westling ^6^

*** Correspondence:** Sophie I. Liljedahl [sophie.liljedahl@vgregion.se](mailto:sophie.liljedahl@vgregion.se)

Supplementary File 2: *The Five Self-Harm Behavior Groupings Measure (5S-HM)*

**Formulation of Five Self-Harm Behavior Groupings:
A Measure (5S-HM)**

Deliberate self-harm refers to complex and multi-determined behaviors. Measurement instruments exist that query non-suicidal self-injury (Gratz, 2001), as well as self-harm regardless of suicidal intent (Hawton, Rodham, Evans & Weatherall, 2002). Research has broadly examined indirect forms of self-harm (St. Germain & Hooley, 2012), as well as frequency and function of direct self-harm behaviors (e.g., Klonsky & Glenn, 2009). To the best of our knowledge and the extent of our literature review (2018), no published measurement instrument examines all of these domains of self-harming behavior concurrently.

The five self-harm across five behavior groupings (5S-HM) was developed to bridge the behavioral gaps in existing self-harm measurement instruments. It is based on the self-harm research literature as well as the clinical observations of the authors that self-harm can change form and function for the same individual both rapidly and over time. For the unified theoretical model underlying this measurement instrument, please see Liljedahl and Westling (2014). Finally, neither this self-harm measure nor the model it is based upon is intended to replace other work based on other self-harm definitions. Rather, they can be used to investigate a number of possibly related clinical syndromes and behavioral phenomena within one instrument.

**Administration**: The 5S-HM is intended for use in clinical settings **with any individual receiving mental health services** **or as part of an assessment to determine whether mental health services may be needed**. If a paper version is used it should be in interview format, not as a self-report measure for participants to complete on their own If it is administered on-line then prompts to skip potentially triggering items must be present (before sexual behavior items and before suicidal behavior items). **The interviewer should be a licensed mental health professional with at least one year of experience with self-harming individuals**.

Administration **can be** **repeated every 14 days**. **Bolded text is read aloud to the participant. Domain and total scores are summed after the interview is administered.**

**For internet administration: If you are aware that you have harmed yourself or created situations where harm to you was a likely outcome, and this has happened more than two times in the last 6 months, please complete the 5S-HM. Otherwise please do not.**

The measure is developed for adults **age 18 years and older** but may be useful for treatment planning for **adolescents seeking services for self-harm** **and related difficulties** such as substance use, eating disorders, post-traumatic stress disorder and, more broadly, emotion dysregulation. It is should not be administered to community/school-based samples or individuals with developmental or autistic-spectrum disorders.

**DO NOT SCORE** before becoming thoroughly familiar with the Appendix, which details inclusion and exclusion behaviors. **The results of this assessment should not be used to predict or assess suicide risk**

**References**

American Psychiatric Association, (2013). Diagnostic and statistical manual of mental disorders (5^th^ ed.). Arlington, VA: American Psychiatric Publishing.

Gratz, K. L. (2001). Measurement of deliberate self-harm: Preliminary data on the Deliberate Self-Harm Inventory. *Journal of Psychopathology and Behavioral Assessment, 33,* 253-263.

Hawton, K., Rodham, K., Evans, E., & Weatherall, R. (2002). Deliberate self harm in adolescents: self report survey in schools in England. *British Medical Journal, 325,* 1207-1211.

Klonsky, E. D., & Glenn, C. R. (2009). Assessing the functions of non-suicidal self-injury: Psychometric properties of the Inventory of Statements about Self-Injury (ISAS). *Journal of Psychopathological Behavior Assessment, 31,* 215-219.

Liljedahl, S. & Westling, S. (2014). A unified theoretical framework for understanding suicidal and self-harming behavior: Synthesis of diverging definitions and perspectives. *Proceedings from the 3^rd^ International Conference on Borderline Personality Disorder and Allied Disorders*: Rome.

St. Germain, S. A., & Hooley, J. M. (2012). Direct and indirect forms of non-suicidal self-injury: Evidence for a distinction. *Psychiatry Research, 197,* 78-84.

The Five Self-Harm Behavior Groupings Measure (5S-HM)

**The following questions ask about behaviors and activities that many people engage in at some point in their lives. I would like to ask you specifically about the last two weeks. Over the last two weeks, have you ever:**

| **1A. Deliberately broken your skin in any way that was not part of ”body art” like piercing or tattooing? For example did you:** | | | |
| --- | --- | --- | --- |
| 1. Scratch yourself with nails or another tool for this purpose (including biting yourself)  \| _1_ YES \| _2_ NO \| \| --- \| --- \| | | | |
| *If yes, how many times over the last two weeks?* | | | |
| _2_ One time | _4_ 2-3 times | _6_ 4-5 times | _8_ 6 or more times |
|  |  |  |  |
| 1. Carved or punctured your skin  \| _1_ YES \| _2_ NO \| \| --- \| --- \| | | | |
| *If yes, how many times over the last two weeks?* | | | |
| _2_ One time | _4_ 2-3 times | _6_ 4-5 times | _8_ 6 or more times |
|  |  |  |  |
| 1. Cut yourself with a razor, knife, a pair of scissors or broken glass  \| _1_ YES \| _2_ NO \| \| --- \| --- \| | | | |
| *If yes, how many times over the last two weeks?* | | | |
| _2_ One time | _4_ 2-3 times | _6_ 4-5 times | _8_ 6 or more times |
|  |  |  |  |
| 1. Rub anything corrosive into your skin or specific body-part (like household cleaner)  \| _1_ YES \| _2_ NO \| \| --- \| --- \| | | | |
| *If yes, how many times over the last two weeks?* | | | |
| _2_ One time | _4_ 2-3 times | _6_ 4-5 times | _8_ 6 or more times |
|  |  |  |  |
| 1. Burned yourself?  \| _1_ YES \| _2_ NO \| \| --- \| --- \| | | | |
| *If yes, how many times over the last two weeks?* | | | |
| _2_ One time | _4_ 2-3 times | _6_ 4-5 times | _8_ 6 or more times |
| 1. Interfere with the healing or wounds on your skin  \| _1_ YES \| _2_ NO \| \| --- \| --- \| | | | |
| *If yes, how many times over the last two weeks?* | | | |
| _2_ One time | _4_ 2-3 times | _6_ 4-5 times | _8_ 6 or more times |
|  |  |  |  |
| 1. Any other similar behaviors you have used for the same purpose?  \| _1_ YES \| _2_ NO \| \| --- \| --- \| | | | |
| *If yes, how many times over the last two weeks?* | | | |
| _2_ One time | _4_ 2-3 times | _6_ 4-5 times | _8_ 6 or more times |
| Please describe what you did: | | | |
| ......................................................................................................................................................... | | | |
| ......................................................................................................................................................... | | | |
| ......................................................................................................................................................... | | | |
| ......................................................................................................................................................... | | | |
| *If any of the above questions were answered with "yes", please also ask the following follow-up questions in relation to # 1A* | | | |
| **Many people describe using these behaviors for different reasons. I am interested in understanding your reasons for using each of these specific behaviors. Although there may be many reasons, please describe the three most important reasons. Rank in order of importance (from most to least important) to me:** | | | |
| Reason (Ranked as number:_____) ......................................................................................................................................................... | | | |
| Reason (Ranked as number:_____) ......................................................................................................................................................... | | | |
| Reason (Ranked as number:_____) ......................................................................................................................................................... | | | |
|  | | | |
| **In the situation(s) you described, was your intent to commit suicide?**   \| _1_ YES \| _2_ NO \| _3_ UNSURE \| \| --- \| --- \| --- \| | | | |
| **1B. Over the last two weeks, did you ever deliberately injure any body part:** | | | |
| 1. By banging your head against a hard surface?  \| _1_ YES \| _2_ NO \| \| --- \| --- \| | | | |
| *If yes, how many times over the last two weeks?* | | | |
| _2_ One time | _4_ 2-3 times | _6_ 4-5 times | _8_ 6 or more times |
|  |  |  |  |
| 1. By banging your arms, legs, hands, feet, or sides against a hard surface to cause a bruise or break a bone?  \| _1_ YES \| _2_ NO \| \| --- \| --- \| | | | |
| *If yes, how many times over the last two weeks?* | | | |
| _2_ One time | _4_ 2-3 times | _6_ 4-5 times | _8_ 6 or more times |
|  |  |  |  |
| 1. Have you deliberately injured any other body part?  \| _1_ YES \| _2_ NO \| \| --- \| --- \| | | | |
| *If yes, how many times over the last two weeks?* | | | |
| _2_ One time | _4_ 2-3 times | _6_ 4-5 times | _8_ 6 or more times |
| Please describe what you did: | | | |
| ......................................................................................................................................................... | | | |
| ......................................................................................................................................................... | | | |
| ......................................................................................................................................................... | | | |
| ......................................................................................................................................................... | | | |
| *If any of the above questions were answered with "yes", please also ask the following follow-up questions in relation to #1B* | | | |
| **Many people describe using these behaviors for different reasons. I am interested in understanding your reasons for using each of these specific behaviors. Although there may be many reasons, please describe the three most important reasons. Rank in order of importance (from most to least important) to me:** | | | |
| Reason (Ranked as number:_____) ......................................................................................................................................................... | | | |

| Reason (Ranked as number:_____) ......................................................................................................................................................... |
| --- |
| Reason (Ranked as number:_____) ......................................................................................................................................................... |
|  |
| **In the situation(s) you described, was your intent to commit suicide?**   \| _1_ YES \| _2_ NO \| _3_ UNSURE \| \| --- \| --- \| --- \| |

| **2A. Over the last two weeks have you ever: Deliberately neglected your physical needs, for example did you:** | | | |
| --- | --- | --- | --- |
| 1. Not take medicine that was prescribed to you (not including forgetting)  \| _1_ YES \| _2_ NO \| \| --- \| --- \| | | | |
| *If yes, how many times over the last two weeks?* | | | |
| _1_ One time | _2_ 2-3 times | _3_ 4-5 times | _4_ 6 or more times |
|  |  |  |  |
| 1. Not see a doctor for a new or chronic health condition (for example, allow a fever, injury or illness more severe than a cold or flu to persist for days or weeks without required medical attention)  \| _1_ YES \| _2_ NO \| \| --- \| --- \| | | | |
| *If yes, how many times over the last two weeks?* | | | |
| _1_ One time | _2_ 2-3 times | _3_ 4-5 times | _4_ 6 or more times |
|  |  |  |  |
| 1. Did you deliberately go without the sleep you needed for more than three nights in a row (excluding parents of small children or those on a short-term work deadline or in exams):  \| _1_ YES \| _2_ No \| \| --- \| --- \| | | | |
| *If yes, how many times over the last two weeks?* | | | |
| _1_ One time | _2_ 2-3 times | _3_ 4-5 times | _4_ 6 or more times |
|  |  |  |  |
| 1. Misuse prescription or over-the-counter medication (taking too much or too little):  \| _1_ YES \| _2_ NO \| \| --- \| --- \| | | | |
| *If yes, how many times over the last two weeks?* | | | |
| _2_ One time | _4_ 2-3 times | _6_ 4-5 times | _8_ 6 or more times |
| *If any of the above questions were answered with "yes", please also ask the following follow-up questions in relation to #2a* | | | |
| **Many people describe using these behaviors for different reasons. I am interested in understanding your reasons for using each of these specific behaviors. Although there may be many reasons, please describe the three most important reasons. Rank in order of importance (from most to least important) to me:** | | | |
| Reason (Ranked as number:_____) ......................................................................................................................................................... | | | |
| Reason (Ranked as number:_____) ......................................................................................................................................................... | | | |
| Reason (Ranked as number:_____) ......................................................................................................................................................... | | | |
|  | | | |
| **In the situation(s) you described, was your intent to commit suicide?**   \| _1_ YES \| _2_ NO \| _3_ UNSURE \| \| --- \| --- \| --- \| | | | |

| **2B. Have you deliberately over the past two weeks gone without:** | | | |
| --- | --- | --- | --- |
| i. Food or adequate nutrition?   \| _1_ YES \| _2_ NO \| \| --- \| --- \| | | | |
| *If yes, how many times over the last two weeks?* | | | |
| _1_ One time | _2_ 2-3 times | _3_ 4-5 times | _4_ 6 or more times |
|  |  |  |  |
| ii. Water or other hydrating fluid?   \| _1_ YES \| _2_ NO \| \| --- \| --- \| | | | |
| *If yes, how many times over the last two weeks?* | | | |
| _2_ One time | _4_ 2-3 times | _6_ 4-5 times | _8_ 6 or more times |

| *If any of the above questions were answered with "yes", please also ask the following follow-up questions in relation to #2B* |
| --- |
| **Many people describe using these behaviors for different reasons. I am interested in understanding your reasons for using each of these specific behaviors. Although there may be many reasons, please describe the three most important reasons. Rank in order of importance (from most to least important) to me:** |
| Reason (Ranked as number:_____) ................................................................................................................................... |
| Reason (Ranked as number:_____) ................................................................................................................................... |
| Reason (Ranked as number:_____) ................................................................................................................................... |
|  |
| **In the situation(s) you described, was your intent to commit suicide?**   \| _1_ YES \| _2_ NO \| _3_ UNSURE \| \| --- \| --- \| --- \| |

| **I am going to ask you questions related to sexual self-harm. Have you in the last two weeks used sexual self-harming behavior, that is, engaged in sexual activity without interest, curiosity or lust but rather for the purpose of harming yourself?**   \| _1_ YES \| _2_ NO (**Skip to #4A*.***) \| \| --- \| --- \|   **Do you think you will be triggered by answering questions about these behaviors or situations?**   \| _2_ No, I do not think I will be triggered. \| _1_ Yes, I think I might be triggered. (**Skip to #4A)** \| \| --- \| --- \| |
| --- | --- | --- | --- | --- |

| **3. Over the last two weeks have you deliberately harmed yourself through sexual behavior? For example, did you:** | | | |
| --- | --- | --- | --- |
| 1. Deliberately have sex despite not wanting to and with the skills/ability to say no (with a partner who you feel confident would have stopped without consequence if you had said no?)  \| _1_ YES \| _2_ NO \| \| --- \| --- \| | | | |
| *If yes, how many times over the last two weeks?* | | | |
| _2_ One Time | _4_ 2-3 times | _6_ 4-5 times | _8_ 6 or more times |
|  |  |  |  |
| 1. Have multiple sex partners on one occasion as an expression of self-harm (not due to curiosity or interest in the experience or for pleasure)  \| _1_ YES \| _2_ NO \| \| --- \| --- \| | | | |
| *If yes, how many times over the last two weeks?* | | | |
| _2_ One time | _4_ 2-3 times | _6_ 4-5 times | _8_ 6 or more times |
|  |  |  |  |
| 1. Have unprotected sex with stranger(s) without asking about sexual health?  \| _1_ YES \| _2_ NO \| \| --- \| --- \| | | | |
| *If yes, how many times over the last two weeks?* | | | |
| _2_ One time | _4_ 2-3 times | _6_ 4-5 times | _8_ 6 or more times |
|  |  |  |  |
| 1. Have unprotected sex with partner(s) known to have sexually transmitted infections for the purpose of contracting the infections?  \| _1_ YES \| _2_ NO \| \| --- \| --- \| | | | |
| *If yes, how many times over the last two weeks?* | | | |
| _4_ One time | _8_ 2-3 times | _12_ 4-5 times | _16_ 6 or more times |
|  |  |  |  |
| 1. Was there any other way you used sexual behaviors (even indirectly for example online or through communication by mobile phone) to harm yourself over the last two weeks?  \| _1_ YES \| _2_ NO \| \| --- \| --- \| | | | |
| *If yes, how many times over the last two weeks?* | | | |
| _2_ One time | _4_ 2-3 times | _6_ 4-5 times | _8_ 6 or more times |

| If yes, can you describe how?: |
| --- |
| ......................................................................................................................................................... |
| ......................................................................................................................................................... |
| ......................................................................................................................................................... |
| ......................................................................................................................................................... |
| *If any of the questions above have been answered with ”yes,” please ask the following questions in relation to #3* |
| **Many people describe using these behaviors for different reasons. I am interested in understanding your reasons for using each of these specific behaviors. Although there may be many reasons, please describe the three most important reasons. Rank in order of importance (from most to least important) to me:** |
| Reason (Ranked as number:_____) ......................................................................................................................................................... |
| Reason (Ranked as number:_____) ......................................................................................................................................................... |
| Reason (Ranked as number:_____) ......................................................................................................................................................... |
|  |
| **In the situation(s) you described, was your intent to commit suicide?**   \| _1_ YES \| _2_ NO \| _3_ UNSURE \| \| --- \| --- \| --- \| |

| **4A. Over the last two weeks, have you ever deliberately put yourself in harms’ way? For example, did you:** | | | |
| --- | --- | --- | --- |
| 1. Walk into a busy street without checking traffic for oncoming vehicles (extreme jaywalking)  \| _1_ YES \| _2_ NO \| \| --- \| --- \| | | | |
| *If yes, how many times over the last two weeks?* | | | |
| _1_ One time | _2_ 2-3 times | _3_ 4-5 times | _4_ 6 or more times |
|  |  |  |  |
| 1. Pick or provoke an extremely uneven physical fight to put yourself in harms’ way (in other words, not as a test of physical strength?)  \| _1_ YES \| _2_ NO \| \| --- \| --- \| | | | |
| *If yes, how many times over the last two weeks?* | | | |
| _2_ One time | _4_ 2-3 times | _6_ 4-5 times | _8_ 6 or more times |
|  |  |  |  |
| 1. Walk alone in neighbourhoods known to be unsafe due to street violence as a method of exposing yourself to risk?  \| _1_ YES \| _2_ NO \| \| --- \| --- \| | | | |
| *If yes, how many times over the last two weeks?* | | | |
| _1_ One time | _2_ 2-3 times | _3_ 4-5 times | _4_ 6 or more times |
|  |  |  |  |
| 1. Use substances known to be associated with extremely adverse outcomes to put yourself in harms’ way?  \| _1_ YES \| _2_ NO \| \| --- \| --- \| | | | |
| *If yes, how many times over the last two weeks?* | | | |
| _2_ One time | _4_ 2-3 times | _6_ 4-5 times | _8_ 6 or more times |
|  |  |  |  |
| 1. Contact or involve yourself in organized crime to put yourself in harms’ way?  \| _1_ YES \| _2_ NO \| \| --- \| --- \| | | | |
| *If yes, how many times over the last two weeks?* | | | |
| _1_ One time | _2_ 2-3 times | _3_ 4-5 times | _4_ 6 or more times |

| 1. Were there any other behaviors you used to put yourself in harms’ way?  \| _1_ YES \| _2_ NO \| \| --- \| --- \| | | | |
| --- | --- | --- | --- | --- | --- |
| *If yes, how many times over the last two weeks?* | | | |
| _1_ One time | _2_ 2-3 times | _3_ 4-5 times | _4_ 6 or more times |
| If yes, please describe: | | | |
| ......................................................................................................................................................... | | | |
| ......................................................................................................................................................... | | | |
| ......................................................................................................................................................... | | | |
| ......................................................................................................................................................... | | | |
| *If any of the above questions were answered with "yes", please also ask the following questions in relation to #4A* | | | |
| **Many people describe using these behaviors for different reasons. I am interested in understanding your reasons for using each of these specific behaviors. Although there may be many reasons, please describe the three most important reasons. Rank in order of importance (from most to least important) to me:** | | | |
| Reason (Ranked as number:_____) ......................................................................................................................................................... | | | |
| Reason (Ranked as number:_____) ......................................................................................................................................................... | | | |
| Reason (Ranked as number:_____) ......................................................................................................................................................... | | | |
|  | | | |
| **In the situation(s) you described, was your intent to commit suicide?**   \| _1_ YES \| _2_ NO \| _3_ UNSURE \| \| --- \| --- \| --- \| | | | |

| **The next set of questions explicitly ask about violent self-harming and suicidal behaviors. Have you engaged in these types of behaviors over the past two weeks?**   \| _1_ YES \| _2_ NO (**Interview complete**) \| \| --- \| --- \|   **Do you think you will be triggered by answering questions about these behaviors?**   \| NO, I do not think I will be triggered. \|  Yes, I think I will be triggered **(Interview complete)** \| \| --- \| --- \| |
| --- | --- | --- | --- | --- |

| **4B. Over the last two weeks have you ever put yourself in harms’ way more directly? For example, did you:** | | | |
| --- | --- | --- | --- |
| 1. Swallow non-ingestible objects or substances (for example, sharp objects or abrasive materials?)  \| _1_ YES \| _2_ NO \| \| --- \| --- \| | | | |
| *If yes, how many times over the last two weeks?* | | | |
| _4_ One time | _8_ 2-3 times | _12_ 4-5 times | _16_ 6 or more times |
|  |  |  |  |
| 1. Try to choke, strangle or hang yourself?  \| _1_ YES \| _2_ NO \| \| --- \| --- \| | | | |
| *If yes, how many times over the last two weeks?* | | | |
| _8_ One time | _16_ 2-3 times | _24_ 4-5 times | _32_ 6 or more times |
|  |  |  |  |
| 1. Engage in extreme risk-taking? For example, did you try to light yourself on fire?  \| _1_ YES \| _2_ NO \| \| --- \| --- \| | | | |
| *If yes, how many times over the last two weeks?* | | | |
| _8_ One time | _16_ 2-3 times | _24_ 4-5 times | _32_ 6 or more times |
|  |  |  |  |
| 1. Did you fire a weapon at your body for the possible risk of killing yourself?  \| _1_ YES \| _2_ NO \| \| --- \| --- \| | | | |
| If yes, which weapon?………………………………………………………………………………………………………………. | | | |
| Which body part? …………………………………………………………………………………………………………………….……….. | | | |
| *If yes, how many times over the last two weeks?* | | | |
| _8_ One time | _16_ 2-3 times | _24_ 4-5 times | _32_ 6 or more times |
| 1. Did you drive in an extremely reckless manner? For example, drunk or well-over the speed limit for the risk of killing yourself?  \| _1_ YES \| _2_ NO \| \| --- \| --- \| | | | |
| *If yes, how many times over the last two weeks?* | | | |
| _4_ One time | _8_ 2-3 times | _12_ 4-5 times | _16_ 6 or more times |
| If yes can you describe what you did?: | | | |
| ......................................................................................................................................................... | | | |
| ......................................................................................................................................................... | | | |
| ......................................................................................................................................................... | | | |
| ......................................................................................................................................................... | | | |
|  | | | |
| 1. For the risk of possibly killing yourself, did you stand, lie, or jump onto train tracks when there was a train approaching?  \| _1_ YES \| _2_ NO \| \| --- \| --- \| | | | |
| *If yes, how many times over the last two weeks?* | | | |
| _8_ One time | _16_ 2-3 times | _24_ 4-5 times | _32_ 6 or more times |

| 1. Was there any other way that you deliberately put yourself directly in harms’ way over the past two weeks?  \| _1_ YES \| _2_ NO \| \| --- \| --- \| | | | |
| --- | --- | --- | --- | --- | --- |
| *If yes, how many times over the last two weeks?* | | | |
| _4_ One time | _8_ 2-3 times | _12_ 4-5 times | _16_ 6 or more times |
| If yes can you describe what you did?: | | | |
| ............................................................................................................................................................ | | | |
| ............................................................................................................................................................ | | | |
| ............................................................................................................................................................ | | | |

| *If any of the above questions were answered with "yes", please also ask the following questions in relation to #4B* |
| --- |
| **Many people describe using these behaviors for different reasons. I am interested in understanding your reasons for using each of these specific behaviors. Although there may be many reasons, please describe the three most important reasons. Rank in order of importance (from most to least important) to me:** |
| Reason (Ranked as number:_____) ........................................................................................................................................................... |
| Reason (Ranked as number:_____) ............................................................................................................................................................ |
| Reason (Ranked as number:_____) ............................................................................................................................................................ |
|  |
| **In the situation(s) you described, was your intent to commit suicide?**   \| _1_ YES \| _2_ NO \| _3_ UNSURE \| \| --- \| --- \| --- \| |

| **5. Over the past two weeks did you ever attempt to end your life?**   \| _1_ YES \| _2_ NO \| \| --- \| --- \| | | | |
| --- | --- | --- | --- | --- | --- |
| *If yes, how many times over the last two weeks?* | | | |
| _8_ One time | _16_ 2-3 times | _24_ 4-5 times | _32_ 6 or more times |
| If yes, what were the specific behaviors?: | | | |
| ......................................................................................................................................................... | | | |
| ......................................................................................................................................................... | | | |
| ......................................................................................................................................................... | | | |
| ......................................................................................................................................................... | | | |
| *If the above question is answered with YES:* | | | |
| **Many people describe using these behaviors for different reasons. I am interested in understanding your reasons for using each of these specific behaviors. Although there may be many reasons, please describe the three most important reasons. Rank in order of importance (from most to least important) to me:** | | | |
| Reason (Ranked as number:_____) ......................................................................................................................................................... | | | |
| Reason (Ranked as number:_____) ......................................................................................................................................................... | | | |
| Reason (Ranked as number:_____) ......................................................................................................................................................... | | | |
|  | | | |
| **In the situation that you have described, was it your aim to take your life?**   \| _1_ JA \| _2_ NEJ \| _3_ UNSURE \| \| --- \| --- \| --- \| | | | |

***Thank you for your time and participation***

**APPENDIX: SCORING SUPPLEMENT
INCLUSION AND EXCLUSION BEHAVIORS**

1. **Self-injury
   Inclusion**: Behaviors in this group range from lower (such as punching, biting or banging ones’ head) to higher severity (such as cutting oneself with a razor knife), and are well-described by non-suicidal self-injury (NSSI) formulations by researchers (e.g., Gratz, 2001) and diagnostic conditions for further study (DSM-5: APA 2013).

**Exclusion i**: Behaviors better accounted for in other four groupings
 **2. Harmful self-neglect
Inclusion**: Behaviors in this group range from not seeking required medical attention for illness or injury, misusing prescription or over-the-counter medication, to going without food or water as a form of deliberately neglecting one’s needs. Deliberately going without adequate sleep for more than 3 nights in a row is also queried

**Exclusion i**: Sleep item for parents of young children, students writing exams, or individuals working on a short-term urgent work deadline.

**3. Self-harming sexual behaviors
Inclusion:** Having unwanted sex as a form of harming oneself, despite feeling capable of saying no, *alongside confidence that one’s partner would have respected the decision not to have sex, without consequence*; having multiple sex partners, not due to curiosity or interest, but as a form of self-harm; having unprotected sex with strangers without asking about sexual health; having unprotected sex with partners known to have sexually transmitted infections (STIs)for the purpose of contracting STIs

**Exclusion i**: Sexual assault.
**Exclusion ii**: Abusive relationships.
**Exclusion iii**: Working in the sex trade and not experiencing this as self-harming or self-exploiting
**Exclusion iv**: BDSM (Bondage/Discipline Sadism/Masochism) and fetishes that are engaged in for pleasure with mutual consent and with safety parameters.
**Exclusion v**: Unprotected sex without the aim to harm one-self or contract HIV or another sexually transmitted infection

1. **Putting oneself in harms’ way
   a. Inclusion for indirect self-harm:** Picking uneven physical fights with an aim to harm oneself rather than test physical strength; walking alone at night in neighbourhoods known to be unsafe for the purpose of exposing oneself to harm; involvement in organized crime for the purpose of exposing oneself to harm.

**b.** **Inclusion for direct self-harm**: Swallowing non-ingestible objects and substances as part of a desire to harm oneself, such as a pencil or liquid bleach, sharp objects or corrosive agents (consistent with Hawton, Rodham, Evans & Weatherall, 2002). Attempting to choke, strangle or hang one-self. Risk-taking behavior with cars, guns, fire, or train-tracks or heights for the purpose of self-harm; Using substances known to be associated with extremely adverse outcomes for the purpose of self-harm.

**Exclusion i:** Abusive relationships
**Exclusion ii:** Physical or sexual assault.
**Exclusion iii**: Playing games in which children, seniors, or animals are provoked or victimized

1. **Suicide attempt
   Inclusion (DSM-V definition extracted from suicide behavior disorder):** “…a self-initiated sequence of behaviors by an individual who, at the time of initiation, expected that the set of actions would lead to his or her own death.” (2013, p. 801)

**Exclusion i:** Is not better explained by self-injury that is non-suicidal in nature and intent
**Exclusion ii**: Suicide attempt does not apply to ideation, planning, or preparing.
**Exclusion iii:** Suicide attempt does not result in completion
