# Supplementary material for: The five self-harm behavior groupings measure: empirical and thematic data from a novel comprehensive self-harm assessment
Source: Front Psychiatry. 2023 May 5;14:1147206. doi: 10.3389/fpsyt.2023.1147206 (PMC10196393; doi:10.3389/fpsyt.2023.1147206)
Supplement: Supplementary file 2 [file Data_Sheet_2.docx]

Supplementary Material

**The Five Self-Harm Behavior Groupings Measure: Empirical and thematic data from a novel comprehensive self-harm assessment**

Sophie I. Liljedahl^1, 2, 3^, Daiva Daukantaité^4^, Nikolaus Kleindienst^5^, Margit Wångby-Lundh^4^, Sofie Westling ^6^

*** Correspondence:** Sophie I. Liljedahl [sophie.liljedahl@vgregion.se](mailto:sophie.liljedahl@vgregion.se)

Supplementary File 2: *List of behaviours encompassed by the 5S-HM classified by directness and indirectness, grouped from low to high lethality*

| Lethality | 5S-HM behaviour grouping | Self-harm type | | Sample item: *“Over the last two weeks have you ever…”* |
| --- | --- | --- | --- | --- |
|  |  | Direct | Indirect |  |
| Low lethality | 1. Self-injury (consistent with non-suicidal self-injury: NSSI) |  |  | **Deliberately broken your skin in any way that was not part of “body art” like piercing or tattooing? For example:** Cutting yourself with a razor, knife, a pair of scissors or broken glass |
|  | 1. Harmful self-neglect |  |  | **Deliberately neglected your physical needs, for example did you:** Misuse prescription or over-the-counter medication (taking too much or too little) |
|  | 1. Sexual self-harm / exploitation |  |  | **Have you deliberately harmed yourself through sexual behaviour? For example, did you:** Deliberately have sex despite not wanting to and with the skills/ability to say no (with a partner who you feel confident would have stopped without consequence if you had said no?) |
|  | 4a. Putting oneself in harms’ way (indirect) |  |  | **Have you deliberately put yourself in harms’ way? For example, did you:** Walk into a busy street without checking traffic for oncoming vehicles (extreme jaywalking) |
| High lethality | 4b. Putting oneself in harms’ way (direct) |  |  | **Have you ever put yourself in harms’ way more directly? For example, did you:** Swallow non-ingestible objects or substances (for example, sharp objects or abrasive materials?) |
|  | 1. Suicide attempt |  |  | **Over the past two weeks did you ever attempt to end your life?**  (Yes / No) If yes, what were the specific behaviours? |
